# Exploring the Intricacies of Finite Element Modeling of 3D-Printed Scaffolds for Musculoskeletal Applications: An In-Depth Review

**DOI:** 10.7759/cureus.101070

**Published:** 2026-01-08

**Authors:** Debangshu Paul, David Sta Maria, SM Anwar Sadat, Md Ataur Rahman, Huma Shahzad, Ehsanul H Apu, Mushfiq H Shaikh

**Affiliations:** 1 Department of Civil and Environmental Engineering, The University of Tennessee, Knoxville, USA; 2 Department of Medical Education, University of Texas Rio Grande Valley School of Medicine, Edinburg, USA; 3 Department of Oral and Maxillofacial Surgery, Dhaka Dental College and Hospital, Dhaka, BGD; 4 Department of Oncology, Karmanos Cancer Institute, Wayne State University, Detroit, USA; 5 Department of Biomedical Sciences, Northeast Ohio Medical University, Rootstown, USA; 6 Department of Dentistry, Bitonte College of Dentistry, Northeast Ohio Medical University, Rootstown, USA

**Keywords:** 3d-printed scaffolds, ai-enhanced methodologies, bone tissue engineering, clinical translation, finite element analysis, multi-scale modeling, patient-specific implants, polymer–ceramic composites, pore architecture, scaffold geometry optimization

## Abstract

Finite element analysis (FEA) is redefining how three-dimensional (3D)-printed bone scaffolds are designed and validated. By digitally predicting stress, strain, and deformation before fabrication, FEA is transforming the field of 3D-printed bone scaffolds by offering a predictive framework to design and validate mechanically robust, biologically active constructs. This review summarizes how FEA-driven strategies optimize scaffold geometry, pore architecture, and material properties, ranging from polymer-ceramic composites to hydrogel blends, under physiological loads. We highlight multiscale modeling approaches that connect microscale porosity to overall strength and discuss live integration of printer feedback for rapid design iterations. Experimental and early clinical validations reveal FEA predictions within single-digit error margins and demonstrate scaffold-guided bone ingrowth in patient-specific implants. Finally, we examine emerging AI-enhanced methodologies for real-time optimization, challenges in modeling degradation and cell remodeling, and propose standardized workflows to accelerate the clinical translation of FEA-informed bioprinted bone scaffolds.

## Introduction and background

Emerging as a transformative technology, the field of three-dimensional (3D) bioprinting has enabled the fabrication of patient-specific scaffolds that intricately replicate the structural and biological nuances of native bone extracellular matrix (ECM) and has achieved a mechanically stable scaffold with high cell viability (>97%) [1]. This technological leap offers unprecedented precision in regenerative medicine, especially for musculoskeletal applications. However, the mechanical reliability under physiological loads has remained a clinical hurdle while translating these innovations into clinical scenarios.

While 3D bioprinting offers greater biological detail and better mimics the in vivo environment, it also presents new challenges, such as scaling and flow limitations of biomaterials [2,3]. Nevertheless, innovative and stimuli-responsive materials, such as electroconductive, self-healing, and shape memory materials, can be incorporated into inks to print even four-dimensional (4D) scaffolds and advanced implantable sensors. Recent research has explored self-healing materials as delivery systems for cells, drugs, biomolecules, and polynucleotides, revealing their potential to aid in tissue regeneration [4]. Due to its multifunctional, electroconductive, and self-healing nature, predicting scaffold growth poses a significant challenge without non-invasive detection techniques [5-7]. The integration between an implant and surrounding tissue is crucial for achieving proper physiological load distribution and successful healing. Post-implantation deformities, such as stress-induced fractures, pore collapse, or shape distortion, compromise scaffold integrity, leading to implant failure or suboptimal tissue integration [8,9]. A significant hurdle for implants is immunogenicity, an immune response triggered by the scaffold's biomaterials (e.g., polymers) or allogeneic (donor) cells, a known risk of traditional grafts. Strategies to prevent this rejection include using autologous (the patient's own) cells, incorporating anti-inflammatory agents, and ensuring high biocompatibility. Biocompatibility is verified through screening tests, such as in vitro and in vivo toxicity assays (ISO 10993). Ideally, these materials are also biodegradable, designed to break down into non-toxic, metabolizable byproducts that the body can eliminate [10].

Finite element analysis (FEA) is a robust numerical method. It divides complex structures into smaller, finite elements (FE). This allows simulation and prediction of their response to physical forces, such as stress, strain, and deformation. FEA is essential in engineering and biomedical research for modeling the mechanical behavior. It provides detailed insights, often beyond the scope of experimental methods. It visualizes internal stress and deformation patterns. This helps optimize design parameters and material properties before fabrication. By modeling how bioprinted scaffolds interact with physical forces, FEA aids in design, material selection, and printing strategies. This is especially important in musculoskeletal applications, as scaffolds must be both mechanically robust and bioactive to support bone regeneration under load. Bone scaffolds must meet both biological and mechanical requirements: they need to support new tissue growth while maintaining structural integrity under load [11]. Bioinks and hydrogel-based materials commonly used in 3D bioprinting are prone to deformation due to low stiffness, swelling, degradation, and physiological stress [12]. However, these materials may pose challenges: biodegradable polymers used in the fabrication of constructs are linked to inflammation, and the clinical implementation of hydrogels has been somewhat restricted due to their limited ability to prevent bacterial colonization, which can trigger an immune response. Key factors influencing post-printing deformation include scaffold geometry, pore architecture, and material properties, such as modulus and viscoelasticity, cellular activity (like remodeling and mineralization), and the specific load conditions in the body [13]. Accurate prediction and control of these deformations are essential for scaffold functionality, and FEA serves as a valuable tool to simulate and optimize scaffold designs before clinical application. Furthermore, research demonstrates that mineralized collagen scaffolds support native-like cellular activity by stimulating osteoblast differentiation and matrix mineralization without the need for supplements, thereby achieving osseointegration that structurally resembles native bone and modulating the immune microenvironment towards a pro-regenerative phenotype [14-16].

This review synthesizes recent advances in FEA-driven design and validation of 3D bioprinted bone scaffolds, emphasizing the coupled roles of biomaterial selection, printing parameters, multiscale modeling, and experimental validation. Bone scaffolds and bone bioinks commonly combine polymers and ceramics to balance bioactivity and mechanical strength, while printing variables such as nozzle diameter, extrusion pressure, and layer height critically regulate pore architecture and stiffness. FEA helps to optimize these trade-offs through virtual parametric exploration of material properties, scaffold geometry, and process conditions to meet competing mechanical and biological targets efficiently [10]. To capture the bone’s hierarchical structure, contemporary FEA frameworks bridge scales from nanoscale collagen and mineral interactions to macroscale (>10 mm) implant mechanics. Micromechanical (1-50 µm) models embed representative volume elements (RVEs) of mineralized collagen fibrils that are parameterized by nanoscale tensile tests within 3D scaffold lattices to capture local stress transfer. RVEs with mesoscale (50 µm-10 mm) lattice simulations, enabling multiscale assessment of failure mechanisms and guiding the design of functionally graded scaffolds that reduce stress shielding and better replicate cortical to cancellous transitions [17-19]. Validation remains central to this workflow; computational predictions are validated using laboratory mechanical testing and imaging under load, allowing simulation models to be refined so they reliably reflect real scaffold behavior in physiological conditions, thereby strengthening their translational relevance for bone regeneration applications [19,20].

By addressing these themes, this article aims to establish a roadmap for leveraging FEA not only as a diagnostic tool but also as a forward-looking strategy to engineer mechanically resilient, biologically functional scaffolds, thereby ushering in a new era of reliability in bone tissue engineering.

## Review

Computational musculoskeletal prediction models

There is a substantial body of literature employing FEA to study the mechanical behavior of human and animal bones. Over the past several decades, FEA has been instrumental in simulating bone strength, predicting fracture risk, and understanding bone mechano-adaptation by incorporating detailed bone geometry, material properties, and physiological loading conditions [21-26]. Studies have shown that FEA can accurately predict endurance limits under cyclic loading conditions. Recent literature has substantiated this capability, demonstrating an exceptional level of accuracy in failure prediction (96.2%) and a strong correlation (R^2^ = 72.6%) between simulation results and experimental data. While early studies focused on density-based predictions, modern FEA, primarily based on high-resolution imaging such as micro-CT, can accurately identify failure regions and optimize orthopedic implant designs for diseases like osteoporosis [27,28]. This predictive ability can be directly applied to tissue engineering, as bioprinted scaffolds are often designed to replicate the complex, anisotropic structure of native bones. Therefore, computational methods that have been proven to understand bone failure mechanisms provide the essential basis for assessing the structural integrity of these printed constructs. The failure behavior of long bones in humans and animals has been extensively studied through experimental and computational approaches, revealing insights into fracture mechanics, load-response dynamics, and material properties [25,26]. There are established frameworks and workflows for computational modeling of hierarchical materials such as bones and 3D-printed scaffolds. These approaches typically integrate multiscale modeling techniques to capture the complex interplay between material properties and structural hierarchy, spanning from the nano- to the macroscale [29,30].

When applied to bioprinted scaffolds, FEA allows researchers to predict how these constructs will behave under physiological loads, optimize scaffold architecture, and identify potential sites of mechanical failure before clinical use [31-33]. By integrating FEA into the design and validation process, the mechanical reliability and functional integration of bioprinted scaffolds can be significantly enhanced, accelerating their translation from laboratory to clinical applications [34]. This capability is especially crucial for musculoskeletal applications, where scaffolds must balance mechanical robustness with bioactivity to support bone regeneration under load-bearing conditions.

FEA analyzes intricate shapes by dividing them into discrete segments, allowing for the prediction of scaffold responses to mechanical and biological factors. In the context of bone scaffolds, FEA enables the simulation of static/dynamic loads modeling physiological forces, such as compression, torsion, and fatigue cycles, to assess endurance limits [35]. It also facilitates deformation prediction by identifying stress concentrations in porous architectures and by accounting for time-dependent viscoelastic creep under sustained loads [36]. Furthermore, FEA supports failure analysis by mapping fracture-prone regions, such as strut junctions in lattice structures, and by assessing degradation-induced weakening [37]. In addition, topology optimization can be achieved by balancing pore geometry, stiffness, and bioactivity through biomimetic designs such as triply periodic minimal surfaces (TPMS) [38].

Constitutive Modeling Approaches

The choice of a constitutive model significantly affects the accuracy of FEA for 3D-printed scaffolds, with each model offering different levels of relevance depending on the fabrication method and material behavior. While linear isotropic elasticity is often used as the default, its applicability to 3D printing is limited unless it employs direct numerical simulation (DNS) to model the exact geometry of each strut; it is generally inaccurate for homogenized models because it does not consider the layer-by-layer nature of the printing process. Conversely, orthotropic elasticity holds significant importance for polymer scaffolds (FDM/SLA), as it embodies the intrinsic anisotropy whereby the printed components are generally less robust and more pliable along the build direction (Z-axis). For soft lattice structures that undergo large bending or buckling prior to failure, hyperelastic models are required to accurately capture nonlinear deformation. Additionally, in bioprinting applications where cells are suspended within a gel matrix, poro-viscoelasticity is essential for simulating critical biological factors such as nutrient diffusion and structural settling. Finally, continuum damage mechanics (CDM) offers specific benefits for failure analysis by enabling researchers to predict fracture points, such as snapping at nodal junctions under load. Different FEA modeling approaches are compared in Table 1.

**Table 1:** Comparison of different FEA modeling approaches FEA: finite element analysis

Model Type	Scaffold Material	Key Assumptions	Limitations
Linear Isotropic Elasticity [39]	Ceramic/titanium lattices (preliminary design)	Material is the same in all directions. Small deformation. No time dependence	Misses the "layering" weakness of 3D printing. Cannot predict failure or flow.
Orthotropic Elasticity [29]	FDM (fused deposition modeling)/SLA (stereolithography) polymers: PCL (polycaprolactone), and PLA (polylactic acid) struts	Stiffness differs in three orthogonal directions	Requires nine independent material constants (hard to calibrate experimentally).
Hyperelasticity [40,41]	Soft tissue scaffolds: TPU (thermoplastic polyurethane), alginate, and hydrogels	Large deformations Non-linear stress-strain curve	Ignores time-dependent effects (creep) and fluid flow.
Poro-Viscoelasticity [42]	Hydrogel-based constructs (bioinks)	Solid matrix + Fluid pores Viscous flow of solid	Extremely computationally expensive. Hard to converge.
Continuum Damage Mechanics (CDM), Phase Field [25,26]	Failure prediction (load-bearing scaffolds)	Stiffness degrades as micro-cracks accumulate (D)	Mesh dependency issues (results change if you refine the mesh).
Non-linear Viscoelasticity (QLV) [43,44]	Soft tissues/elastomers: ligaments, tendons, and silicone	Large deformations. Strain-rate dependent memory effect (relaxation)	Computationally expensive. Hard to separate the viscous effects from the hyperelastic effects during fitting.
Poro-elasticity (Biphasic) [45]	Hydrated tissues: articular cartilage and dense hydrogels	Solid matrix is elastic (spring). Fluid flows through pores (Darcy’s Law) Incompressible constituents	Cannot capture intrinsic matrix relaxation (viscosity). Requires complex contact definitions in FEA.

General Workflow of Computational Modeling

**Figure 1:**
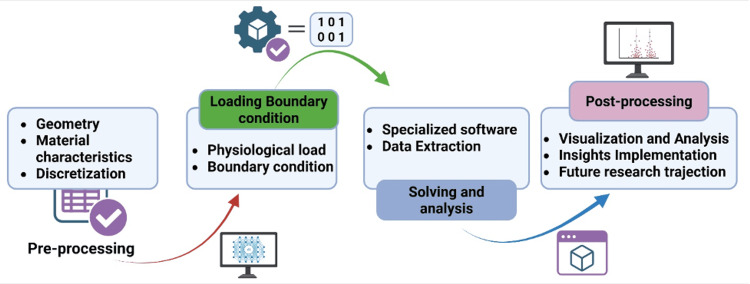
Stepwise framework of computational modeling, from geometry drafting to post-processing. The workflow shows the transition from digital scaffold construction and mesh formulation to loading, solving, and interpretation for biomechanical insight. Figure created by Rahman M (2025) using BioRender (https://BioRender.com/cxrvg9r).

The general workflow of computational modeling follows a structured sequence that begins with preparing the digital representation of the scaffold and extends through simulation, analysis, and post-processing. These steps are illustrated in Figure 1, which highlights the sequential process from geometry drafting to interpretation of results.

Initial preparations (pre-processing): The pre-processing stage begins with drafting the geometry, where the digital twin of the scaffold and the surrounding tissues is created using medical imaging scans and design software, incorporating patient-specific patient anatomy when necessary. This is followed by defining material characteristics, which involves assessing relevant properties of both the bioprinted material and nearby tissues, such as bone, cartilage, and muscle, based on established databases and scholarly literature. Finally, the digital prototype is partitioned into smaller elements or 'meshes,' to simplify and optimize the computational calculations.

Setting loading and boundary conditions: In this stage, physiological loads are applied to the model to simulate everyday activities, such as walking, running, or jumping, thereby replacing everyday mechanical demands on the scaffold. At the time, boundary conditions are specified by defining fixed or restricted areas within the system, ensuring that the simulation accurately reflects the physical constraints encountered in vivo.

System solving and analysis: At this stage, specialized software resolves the system of equations derived from the predefined material properties, geometry, and loading conditions. Once executed, the software generates outputs such as stress levels, strain rates, deformation extents, and other biomechanical parameters at various points within the model, providing a comprehensive assessment of scaffold performance.

Interpreting results (post-processing): In the post-processing phase, results are visually represented and carefully analyzed to understand how the scaffold responds to applied loads. The insights gained from this analysis are then implemented to refine the scaffold design, optimize performance, anticipate potential issues, and direct future research trajectories.

Validation and Experimental Correlation of FEA Predictions

An exceptional level of accuracy of 96.2% has been demonstrated in literature [45]. However, to ensure clinical relevance, these computational predictions must be validated through multimodal experimental approaches.

Mechanical testing: It plays a crucial role in validating FEA predictions. Compression and indentation tests are commonly used to directly compare FEA-predicted stress-strain curves with experimental measurements, ensuring the accuracy of the simulations. In addition, dynamic loading studies, such as fatigue tests on TPMS-based scaffolds, have revealed that FEA can accurately predict endurance limits under cyclic loading conditions.

Digital image and volume correlation (DIC/DVC): DIC is a non-invasive technique utilized for surface-level strain mapping, providing detailed insights into localized deformation patterns and mechanical performance. While DIC efficiently captures strain distribution across the scaffold exterior, it is complemented by micro-CT combined with DVC, which extends the analysis to subsurface regions. This high-resolution 3D visualization enables precise tracking of scaffold deformation, including pore collapse, strut fractures, and morphological changes induced by sintering. This combination offers critical insights that help reconcile discrepancies between surface-level measurements and internal deformation patterns.

Histological analysis: It is performed post-implementation to evaluate scaffold integration within the host environment. This assessment focuses on tissue-scaffold interactions, including cellular infiltration, ECM deposition, and biocompatibility. Common staining methods, such as hematoxylin and eosin (H&E) for general morphology and Masson's trichrome for collagen deposition, are frequently employed to provide detailed insights into the quality and extent of tissue regeneration.

Recent Advances in Coupled FEA Approaches

The fidelity of FEA simulations depends on the selection of appropriate constitutive models and the integration of advanced modeling strategies.

Hierarchical modeling: Integrates micro-CT data to resolve trabecular-scale porosity, enabling precise mapping of stress concentrations and failure points at strut junctions.

Coupled multi-physics (FSI and mechano-biology): Fluid-structure interaction (FSI) models ensure vascularized scaffolds maintain structural stability under hemodynamic flow. These are often paired with mechano-regulation algorithms that predict tissue differentiation and matrix remodeling based on local strain thresholds and nutrient transport.

Machine learning (ML) integration: ML algorithms augment traditional FEA to rapidly predict deformation and optimize scaffold architectures, significantly reducing the computational cost of evaluating large design libraries.

Applications across the musculoskeletal systems

Bone Scaffolds

Cancellous bone provides critical support, cushioning, and resistance to deformation. Still, current treatments for bone defects often rely on autogenous grafts, which are limited in supply and carry risks of infection and surgical complications. Therefore, artificial bone scaffolds are being extensively studied, with porosity and pore size emerging as key design parameters to enhance bone regeneration and mechanical performance. A recent study utilized FEA to analyze porous tricalcium phosphate (TCP)/polycaprolactone (PCL) composite scaffolds, showing that higher porosity reduced compressive strength and triangular pores provided the most outstanding stability. Among the evaluated scaffold geometries, the diamond-shaped design exhibited the lowest stability, while the triangle architecture demonstrated the highest axial compressive strength [46]. The finite element simulations closely aligned with experimental measurements across both triangular and CAD-based wavy and hexagonal models, confirming the reliability of FEA in predicting scaffold mechanical performance [47].

Finite element modeling (FEM) has become a critical tool for characterizing the mechanical behavior of bone-mimetic materials, allowing scaffold performance to be evaluated under physiologically relevant loading conditions and enabling the rational design of tissue-specific scaffolds [48]. By stimulating local stress and strain environments, FEM facilitates optimization of scaffold porosity and geometry to promote osteogenesis while maintaining mechanical integrity [19]. Importantly, computational screening of strain behavior in 3D porous scaffolds has been shown to reduce the need for extensive physical testing and improve design efficiency [49]. From a clinical perspective, conventional strategies for long-bone defect reconstruction, including autografts, allografts, and prosthetic implants, are limited by donor-site morbidity, immune complications, and suboptimal integration [50]. These limitations have driven increasing interest in patient-specific, 3D-printed scaffolds, which provide substantial design flexibility for generating complex, defect-matched porous architectures for bone regeneration. Despite these advances, significant challenges remain. Current additive manufacturing platforms must achieve improved combinations of mechanical strength and low elastic modulus, while advances in computational software need to be developed to appropriately identify the material within 3D bone scaffold models. Furthermore, expanding the range of material and geometry design parameters is important for the optimization of internal architecture and bone scaffold material [10].

Recent modeling efforts have emphasized that incorporating a broader range of input variables improves agreement between virtual simulations and experimental outcomes [51,52]. In this context, Kakarla et al. integrated material designer and RVE analysis and FEM to analyze boron nitride nanotubes (BNNts)-reinforced gelatin and alginate hydrogel, capturing both macroscopic mechanical behavior and microstructural reinforcement effects. Together, these findings demonstrated that advanced materials can be effectively modeled using the FEA and RVE framework, thereby encouraging experimentation with novel bioactive combinations for bone regeneration [52].

Scaffolds for Cartilage Repair

Cartilage exhibits poor regeneration due to a lack of vasculature, nerves, and lymphatics. Despite how common cartilage injuries are, current treatments cannot restore native tissue. Instead, they are focused on relieving symptoms [53,54]. However, the development of 3D-printed constructs for cartilage repair, alongside FEM, is demonstrating promising solutions for cartilage regeneration and offers the potential for seamless scaffold integration with surrounding cartilage that can withstand complex joint stresses while encouraging chondrocyte proliferation and differentiation.

Desirable characteristics of an effective scaffold for cartilage repair include mechanical strength, high biocompatibility, and sufficient water absorption. Ideally, the construction should match the mechanical and biological properties of native extracellular matrices. In addition, various parameters need to be considered when designing such a construction. For instance, porosity is an essential variable that heavily influences the diffusion of nutrients and the degree of vascularization, followed by cell growth. Pore size is also a crucial parameter to consider, as too small a size would constrict the flow of nutrients, while too big a pore size limits the cell adhesion to the construct [54]. To engineer an appropriate bio-construct, FEM allows for analysis of various scaffold parameters to optimize tissue engineering therapy [55, 56]. Utilizing medical imaging, mechano-regulation theory, and FEM, researchers develop a 3D knee joint model that simulates tissue regeneration over time. Through this integrated FE simulation, different scaffold material properties in three depth-based zones were analyzed, including elastic modulus, Poisson's ratio, and permeability. An optimization algorithm identified scaffold conditions that maximized cartilage formation while minimizing cell death and fibrous tissue, achieving 68% regeneration compared to 21% without a scaffold. This was due to the scaffold’s ability to support structure, mimic the ECM, and modulate mechanical environments. When combined with FEA, scaffold design can be further refined. Likewise, another research group utilized FEA to analyze the mechanical properties of scaffolds composed of chitosan-gelatin with fluorinated hydroxyapatite (FHA) nanoparticles [57]. Similarly, in silico models using FEA can simulate mechanical responses under physiological loads [58], helping repair irregular defects that are difficult to treat clinically [53].

Thus, scaffolds developed alongside computational modeling show greater promise than current graft treatments, as they are less invasive and significantly promote cell adhesion and proliferation through modified mechanical loading at the defect site [59]. This optimization of scaffold engineering results in reduced expenses and time spent on experimental testing.

Nevertheless, there are many limitations in FEM and cartilage scaffold engineering. One of them is the lack of compatible artificial cartilage complexes, which can be attributed to poor integration and stagnation of hyaline differentiation. However, FEM allows for the discovery of new compatible artificial cartilage materials. A recent study used a coupled FE-optimization algorithm to show that PVA hydrogels have mechanical properties similar to native cartilage, supported by agreement between predicted and experimental stress-relaxation data [55]. Similarly, 4D bio-constructs are gaining attention for their ability to adapt scaffold properties in response to environmental stimuli [54]. Further research is needed to develop FEMs that fully capture cartilage anisotropy and to understand how cell-scaffold interactions influence cartilage repair outcomes [53]. Overall, more studies are needed to ensure the reproducibility, effectiveness, and safety of bioprinted products for achieving cartilage repair.

Scaffolds for Muscle and Tendon Repair

**Figure 2:**
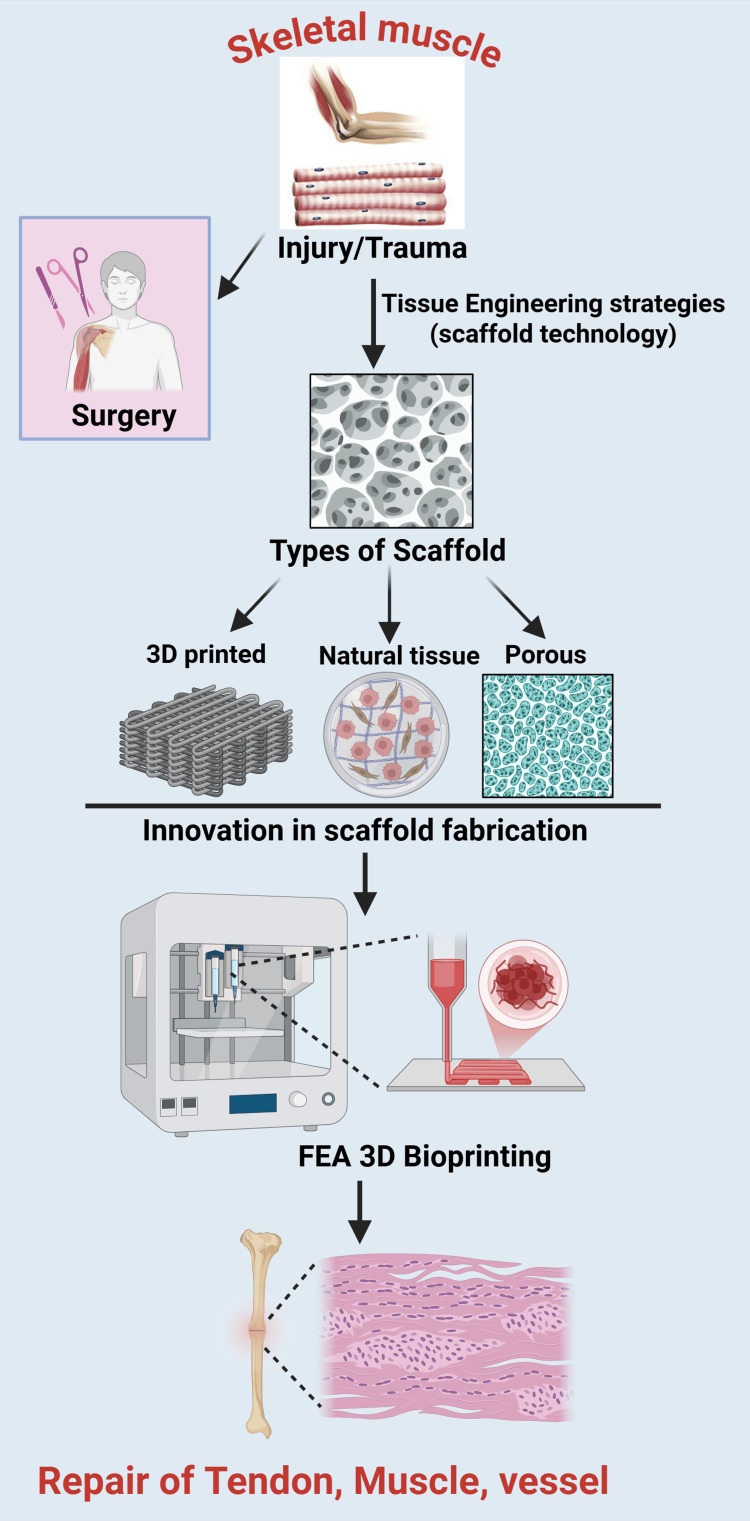
Schematic representation of scaffold-based tissue engineering and finite element analysis (FEA)-assisted 3D printing for musculoskeletal healing. The process starts with injury or stress to skeletal muscle, necessitating surgical intervention. Tissue engineering methodologies utilize diverse scaffold technologies, such as 3D-printed constructions, natural tissue-derived scaffolds, and porous synthetic matrices, to restore function. Progress in scaffold fabrication facilitates the amalgamation of finite element analysis (FEA) with 3D printing, refining scaffold shape, pore architecture, and muscle and vascular tissue materials. Figure created by Rahman M (2025) using BioRender (https://BioRender.com/84cf2sc).

FEA has increasingly been utilized in the field of muscle and tendon repair, with several case studies demonstrating its effectiveness in informing repair techniques and improving outcomes. The overall framework of scaffold-based tissue engineering and the role of FEA in supporting 3D printing for musculoskeletal healing are illustrated in Figure 2. Its application spans several critical areas, including injury mechanics, treatment efficacy, and device optimization. FEA is utilized to model the complex biomechanics of tendons, which consist of hierarchically organized fibrous connective tissues that connect muscles to bones. By simulating tendon behavior under different loading conditions, researchers can predict how tendons will respond to stress and strains, thereby enhancing the understanding of injury mechanisms such as strains and tears [60,61]. This understanding is crucial for developing effective treatment strategies and rehabilitation protocols.

A recent study applied FEA to design piezoelectric scaffolds for tendon repair by predicting mechanical and electrical behavior to match the properties of natural tendon tissue closely [62]. This tailored approach enhances the integration of the scaffold with surrounding tissues, improving the overall healing process. In another case, FEA analysis of homogeneous and gradient scaffolds showed that stiffness gradients enable more uniform load distribution, improving functional outcomes in tendon repair [63]. The results support the notion that optimized scaffold designs can effectively mimic the mechanical properties of natural tendon structures. Furthermore, the utilization of FEA has advanced the design and development of melt electrowiring (MEW)-based 3D tubular scaffolds that mimic tendon mechanics and support customizable architectures for improved stability and regenerative function [64]. Additionally, FEA is applied to assess the performance of various suture materials and configurations, thereby guiding surgeons in choosing the best options to reduce failure rates associated with tendon repairs [65].

Appropriate mechanical loading, guided by FEA, improves tendon regeneration by optimizing conditions that promote collagen production and structural integrity while reducing the risk of re-injury. One of the primary advantages of FEA is its ability to simulate the mechanical behavior of biological tissues under various loading conditions, which helps predict potential complications or failures during surgical procedures [66]. This predictive capability is critical in planning interventions, as it allows surgeons to foresee issues such as stress concentrations, strains, and potential failure points, ultimately enhancing patient safety and outcomes. The analyzing factors, such as graft size, tunnel geometry, and fixation methods in anterior cruciate ligament reconstruction (ACLR), can inform best practices and improve clinical guidelines based on simulated outcomes [61,66,67]. As the FEA continues to advance, its contributions to orthopedic research are becoming increasingly significant. It has fostered a deeper understanding of the mechanics of muscle and tendon injuries, as well as the effects of different surgical interventions, paving the way for innovative treatment options and enhanced recovery protocols [63,66].

With the ongoing research advancements, the future of FEA in muscle and tendon repair is expected to involve the integration of artificial intelligence (AI), advancing computational models, and applying novel scaffold materials to enhance predictive accuracy and therapeutic outcomes. By utilizing ML algorithms, researchers can improve the accuracy of predictive models, optimize scaffold designs, and enhance real-time monitoring during tissue regeneration processes. For instance, AI can facilitate the optimization of biomaterials for scaffolds, customize 3D-bioprinted tissues, and standardize disease models, ultimately leading to better patient outcomes in regenerative therapies [68,69]. However, challenges such as high costs and ethical concerns must be addressed to enable broader adoption of these technologies in clinical settings [69]. The development of advanced computational models is another crucial direction for future research. Utilizing techniques such as topology optimization, researchers can create scaffolds that closely mimic the natural architecture of tendons, promoting better integration and function post-surgery. In addition, multiscale modeling can help simplify complex physiological environments and enhance understanding of anatomical interactions, aiding the development of more effective repair strategies [70]. Future research will likely focus on optimizing biomaterials and bioinks, particularly those combining decellularized ECM with polymers, to improve biocompatibility, reduce immunogenicity, and support tissue regeneration [69].

Neurovascular Regeneration

Neurovascular regeneration refers to the complex processes involved in the repair and regeneration of neural tissues and their associated vascular networks. Mechanical signals are known to influence cell differentiation and tissue development, both of which are pivotal to neurovascular regeneration. For instance, mechanical stimuli, such as fluid shear stress and compressive loading, can activate mechanoreceptors on cells, thereby triggering signaling pathways that facilitate tissue repair and regeneration [19,71]. The application of mechanical loading through bioreactor systems has been demonstrated to enhance the osteogenic development of human mesenchymal stem cells, which is essential for bone regeneration [71]. Recent studies emphasize the interdependence between biomaterials and biological systems. The modification of biomaterials' physical properties and structural characteristics can significantly impact cellular behavior, including migration, proliferation, and differentiation. This interaction is crucial for designing scaffolds that effectively support neurovascular regeneration by mimicking the natural ECM and promoting the necessary biological responses [17,72].

The practical design of scaffolds plays a vital role in improving regenerative medicine therapies. 3D-printed scaffolds are essential components in the field of tissue engineering, particularly for neurovascular regeneration. In the context of neurovascular applications, specific bioink compositions have been explored for their ability to enhance cell viability and differentiation within 3D-printed scaffolds. Most studies in this area emphasize key factors in scaffold design that impact cellular behavior but provide limited examples of specific bioink formulations. In particular, biocompatibility and biodegradability are paramount for scaffolds, ensuring that the materials do not elicit adverse responses and can be appropriately integrated into biological systems [73,74]. Additionally, pore interconnectivity, pore size, and porosity are essential characteristics that influence nutrient flow and cellular migration, ultimately affecting cell viability and differentiation [75]. Furthermore, the mechanical properties of bioinks, which are influenced by their composition and concentration, can play a significant role in the performance of the scaffolds. A study highlighted that different infill patterns of polylactic acid (PLA) scaffolds demonstrated substantial variations in mechanical strength, which could correlate with cellular responses [76]. The right mechanical properties can promote a suitable microenvironment for cell attachment, proliferation, and differentiation [77].

Tailoring the scaffold design through computer-aided design (CAD) and FEM allows researchers to predict and optimize mechanical behaviors, enhancing the functionality of the scaffolds. This is evident by a study that demonstrated the effective use of FEM in the design and optimization of 3D-printed scaffolds for neurovascular regeneration and analyzed the mechanical properties of scaffolds under load-bearing conditions, leading to improved scaffold performance and biocompatibility [78]. One notable case involved the use of FEM to optimize scaffold geometry and material properties for specific tissue types, such as bone and vascular structures. By simulating in vitro and in vivo loading conditions, researchers could refine the design of scaffolds to achieve superior stress distribution and enhance cell viability [76]. Therefore, the physical characteristics of 3D-printed scaffolds, including mechanical properties, bioink composition, and printing parameters, significantly influence cellular behavior, facilitating interactions essential for successful neurovascular regeneration.

Despite the advancements in the field, several challenges remain in achieving effective neurovascular regeneration. These include the complexity of simulating the physiological conditions of human tissues, the need for precise control over the scaffold architecture, and the requirement for multidisciplinary approaches to integrate various fields, such as computational modeling, biomaterials science, and cellular biology. Emerging technologies such as 3D printing and four-dimensional (4D) printing strategies offer promising avenues for overcoming these challenges by enabling the creation of dynamic scaffolds that can adapt to physiological changes and support both neural and vascular regeneration [78,79].

Additionally, challenges remain in translating FEM-informed designs from preclinical studies to clinical applications. To address these challenges, researchers recommend enhancing preclinical testing through improved animal models and implementing adaptive clinical trial designs that respond dynamically to evolving data [72]. Such strategies could improve the safety and efficacy of scaffold applications in human trials, paving the way for innovative treatments in neurovascular regeneration [72]. Moreover, a concerted effort towards interdisciplinary collaboration between computational scientists and biological researchers could foster creative solutions and accelerate advancements in the field.

Challenges and future directions

Challenges

Model accuracy: Achieving high model accuracy remains a significant challenge, as it requires capturing the intricate behavior of living cells, biomaterials, and the dynamic interaction within the musculoskeletal system. Incorporating complex factors, such as cell migration, differentiation, and tissue remodeling and potential immunogenic responses leading to rejection [80-82], requires advanced approaches and remains an area of ongoing research. Decellularized ECM scaffolds offer the elastic architecture essential for lymphatic drainage; however, they pose considerable immunogenic risks, including graft-versus-host disease, when not HLA-matched. Conversely, synthetic hydrogels utilize adjustable biomechanical properties to guide cellular function, wherein specific tissue stiffness levels (e.g., approximately 2000 Pa) serve as mechanical stimuli to surface receptors, thereby regulating cell proliferation.

Computational power and complexity: Modeling large and complex tissue structures with sophisticated material properties can be computationally demanding, requiring high-performance computing resources. To achieve faster and more realistic simulations, efficient computational tools and algorithms are essential. The challenge is further compounded by nonlinear analyses, such as viscoelasticity and contact mechanics, which significantly increase runtime, particularly when dealing with large-scale or patient-specific models [43,83]. To accurately capture the behavior of hybrid biomaterials, current computational frameworks are increasingly moving beyond simple isotropic assumptions. For the structural phase, orthotropic elasticity models are employed to characterize the direction-dependent stiffness of 3D-printed polymer struts. At the same time, poroviscoelastic formulations are utilized for the hydrogel matrix to account for fluid and solid interactions and time-dependent viscous flow. Furthermore, advanced composite models now integrate these distinct phases to simulate complex architectures, such as collagen fiber-reinforced hydrogels, enabling the prediction of how anisotropic topographical cues influence both mechanical stability and cellular guidance.

Clinical translation: Bridging the gap between in silico predictions and real-world clinical outcomes is crucial for realizing the full potential of FE modeling in scaffold design. Reliable clinical translation requires thorough validation through *in vitro* and *in vivo* experiments, combined with well-structured clinical trials to confirm efficacy and safety. Nonetheless, exclusive reliance on conventional biological validation introduces significant reproducibility challenges. For example, animal models often lack physiological relevance to human biomechanics, resulting in clinical translation success rates below 8% in certain therapeutic areas. Moreover, the considerable time and financial investments required for regulatory approval, often exceeding a decade and costing billions of dollars, emphasize the need to adopt high-fidelity FE modeling to establish standardized validation protocols and expedite market entry [81]. Establishing clear regulatory pathways for personalized medicine applications that incorporate FE-designed scaffolds is also critical. Additionally, the use of advanced imaging modalities such as micro-CT, CT scans, and MRI is vital for accurately analyzing the fracture/stress locations and stress distributions.

Temperature maintenance: Maintaining and accurately modeling physiological temperature effects during both bioprinting and in vivo conditions is challenging; temperature fluctuations can influence material properties, degradation rates, and mechanical performance, yet are often neglected in current FEA models. For instance, computational fluid dynamics (CFD) simulations have demonstrated that deviations in bioink temperature significantly alter viscosity and wall shear stress, leading to unstable extrusion pressures and velocities that compromise print fidelity [84]. Furthermore, the thermal history of the material directly influences microstructural characteristics, such as polymer chain distribution and porosity, which are critical determinants of the scaffold's swelling behavior and long-term degradation kinetics in physiological environments [85].

Limited mechanical data for novel bioinks: Many emerging bioinks (e.g., decellularized matrix hydrogels, conductive polymers) lack standardized mechanical characterization under in vivo conditions (hydration, temperature, dynamic loading). Anisotropic or time-dependent behaviors (e.g., shearthinning, swelling) are often unaccounted for, forcing idealized assumptions in simulations. Addressing the multifaceted challenges of model accuracy, computational expense, and clinical translation necessitates a paradigm shift toward integrative technologies. The subsequent future directions employ advanced methodologies, such as multiscale simulation and AI-driven optimization, not merely as enhancements but as direct solutions to the bottlenecks above. By harnessing these innovations to enhance material characterization and automate intricate analyses, the discipline can effectively bridge the divide between theoretical FE modeling and clinically viable, practically applicable bioprinted constructs.

Future Directions

Multiscale modeling: Integrating FE models with other computational tools like molecular simulations and ML will offer a more comprehensive understanding of scaffold performance at various scales, from the cellular level to the entire tissue construct.

Patient-specific modeling: Advancements in medical imaging and data analysis will enable the creation of even more accurate and personalized FE models, leading to truly individualized scaffold designs and improved treatment outcomes. Beyond structural optimization, FE modeling also supports the development of multifunctional scaffolds that incorporate additional functionalities, such as drug delivery or electrical stimulation, for enhanced therapeutic efficacy. Looking ahead, integrating FE modeling with real-time monitoring of bioprinted constructs and patient-specific data could establish closed-loop systems for continuous real-time optimization of scaffold design and treatment strategies. Utilization of CFD and FSI can facilitate the simulation of blood flow, nutrient transport, and mechanical cues within bioprinted scaffolds. By embedding patient-derived vascular networks and hemodynamic data into FEA enables tailored designs that optimize perfusion, shear stress thresholds, and neurovascular integration while mitigating deformation risks.

AI-powered scaffold optimization: Leveraging AI to automate and accelerate scaffold design, enabling rapid identification of optimal architectures and material combinations. Furthermore, establishing uniform testing methods to generate reliable, comparable mechanical data across scaffold materials and designs. Additionally, integration of patient-specific imaging, such as MRI and CT data, can be utilized to create highly personalized scaffold models, enhancing the fit and functional outcomes.

## Conclusions

FEM has emerged as a critical tool in advancing 3D bioprinted musculoskeletal systems by enabling rational design, optimization, and personalization of scaffolds tailored to patient-specific anatomical and mechanical needs. Future progress will rely on closer integration of FEM with AI designing tools and advanced bioprinting hardware, creating efficient 'digital-to-physical' workflows that accurately convert medical imaging data into functional bioprinting constructs. Equally critical to clinical translation is the development of standardized validation and regulatory pathways, which could reduce dependence on extensive animal testing and streamline approval processes. Achieving these goals will require sustained interdisciplinary collaboration among engineers, materials scientists, biologists, and clinicians to ensure that FE-guided bioprinting solutions are both scientifically robust and clinically practical. As research continues to push the boundaries of FEM, the future of musculoskeletal repair and regeneration holds immense promise for personalized medicine and a healthier future for all.
